# Pathological features and genetic alterations in colorectal carcinomas with characteristics of nonpolypoid growth

**DOI:** 10.1038/sj.bjc.6601965

**Published:** 2004-06-22

**Authors:** K Kaneko, T Kurahashi, R Makino, K Konishi, H Ito, A Katagiri, Y Kumekawa, Y Hirayama, K Yoneyama, M Kushima, M Kusano, H Tajiri, B J Rembacken, K Mitamura, M Imawari

**Affiliations:** 1 Second Department of Internal Medicine, Tokyo, Japan; 2 Clinical Research Laboratory, Tokyo, Japan; 3 Department of Pathology, Tokyo, Japan; 4 Second Department of Surgery, Showa University School of Medicine, Tokyo, Japan; 5 Department of Endoscopy, Jikei University School of Medicine, Tokyo, Japan; 6 Centre for Digestive Diseases, The General Infirmary, Leeds, UK

**Keywords:** colorectal carcinoma, PCR–SSCP, genetic pathway, adenoma–carcinoma sequence

## Abstract

We sought to clarify pathological features and genetic alterations in colorectal carcinomas with characteristics of nonpolypoid growth. Colorectal carcinomas resected at Showa University Hospital in Tokyo included 86 with characteristics of polypoid growth (PG) and 21 with those of nonpolypoid growth (NPG). Mutations of APC, Ki-*ras*, and p53 genes, as well as microsatellite instability (MSI), were analysed using fluorescence-based polymerase chain reaction–single-strand conformation polymorphism (PCR–SSCP). Carcinomas with an NPG pattern were smaller than PG tumours (*P*<0.0001). Carcinomas with a PG pattern were more likely to harbour Ki-*ras* mutations (36%) than NPG tumours (0%; *P*<0.0001). Mutation types in the APC gene differed significantly between PG and NPG carcinomas (*P*=0.0189), including frameshift mutations in 66% of PG carcinomas but no NPG carcinomas. Presence of a p53 mutation at a ‘hot spot’ also was more likely in PG carcinomas (37%) than in NPG carcinomas (0%; *P*=0.0124). No significant difference in presence of MSI was evident between carcinomas with PG and NPG patterns. In conclusion, significant genetic differences were evident between carcinomas with PG and NPG patterns. Genetic changes in NPG carcinomas differed from those of the conventional adenoma–carcinoma sequence. Assuming that some nonpolypoid growth lesions transform rapidly into advanced carcinomas, 20% of all colorectal carcinomas may progress in this manner.

The distinction between flat or depressed colorectal cancers and polypoid colorectal cancers now is well accepted endoscopically. However, the frequency of colorectal carcinomas originating from a flat or depressed (nonpolypoid) precursor lesion is unknown. Two pathways for development of colorectal carcinomas have been proposed, one being the adenoma–carcinoma sequence by which many carcinomas develop from polypoid adenomas (Morson, 1968; Muto *et al*, 1975), and the other involving essentially flat precursor lesions, often with a central depressed area. Histologically, flat lesions are more likely to harbour high-grade dysplasia even when small (Muto *et al*, 1985). Furthermore, some depressed cancers may develop *de novo* (Kuramoto and Ohara, 1988; Kudo, 1993).

In 1990, a multistep genetic model for colorectal tumorigenesis was proposed (Vogelstein *et al*, 1988; Fearon *et al*, 1990; Fearon and Vogelstein, 1990), in which genetic alterations involving an oncogene and several tumour suppressor genes accumulate in the tumour cell lineage. APC gene mutations are involved in the initial step of adenoma formation, while Ki-*ras* mutations make adenomas larger and more severely dysplastic and p53 mutations promote malignant transformation (Bos *et al*, 1987; Vogelstein *et al*, 1988; Baker *et al*, 1989; Fearon *et al*, 1990; Fearon and Vogelstein, 1990; Miyoshi *et al*, 1992; Powell *et al*, 1992). In another genetic pathway to colorectal neoplasia, microsatellite instability (MSI) is caused by mutations in nucleotide mismatch repair genes (Marra and Boland, 1995; Kinzler and Vogelstein, 1996). MSI is characterised by additions and deletions of nucleotides in numerous repeated nucleotide sequences (microsatellites). Patients with MSI-positive colorectal carcinoma have better survival (Hemminki *et al*, 2000). Alteration of a 10-base pair (bp) polyadenine tract in the transforming growth factor-*β* type II receptor (TGF-*β* RII) gene is present in many MSI-positive carcinomas (Parson *et al*, 1995; Akiyama *et al*, 1997). While the APC, Ki-*ras*, and p53 genes, as well as MSI, have been characterised with regard to carcinogenesis in polypoid adenomas, a specific gene responsible for carcinogenesis in flat adenomas has not yet been identified.

We encountered a patient in whom a minute depressed lesion previously had been missed in interpretation of barium enema radiographs; it transformed rapidly into an advanced carcinoma with nonpolypoid growth (NPG). In previous studies, some minute nonpolypoid neoplasias have been reported to transform to nonpolypoid cancers manifesting rapid growth (Minamoto *et al*, 1994; Matsumoto *et al*, 1995; Fujiya and Maruyama, 1997). The central depression has been reported to be clearly delineated radiographically in 32 out (60.4%) of 53 nonpolypoid neoplasias measuring 5 mm and less (Fujiya and Maruyama, 1997). Matsumoto *et al* (1993) have shown that barium enema examination can demonstrate small flat and depressed adenomas of the colon, although colonoscopy seems superior to barium enema examination for detection of these lesions. The previous barium enema radiograph indicated depressed area of the tumour alone, but no elevated portion surrounding depressed area was found in our case. The histologically entire size of the tumour has been shown to correspond to the size of the depressed area in barium enema examination (Matsumoto *et al*, 1993; Minamoto *et al*, 1994; Watari *et al*, 1997). Thus, the advanced carcinoma in our case was thought to have truly derived from a minute depressed-type lesion. If flat lesions containing depressed areas progress rapidly, investigation of the frequency, morphologic characteristics, and genetic alterations of consequent carcinomas is particularly important. Flat or depressed lesions are very difficult to identify by conventional diagnostic techniques (Fujii *et al*, 1998; Rembacken *et al*, 2000); if they develop through distinct genetic pathways, genetic diagnostic approaches to screen for this type of colorectal neoplasia might provide more early detection. Assuming that transformation of flat lesions containing depressed lesions may be rapid, we investigated the genetic makeup of carcinomas originating from flat lesions with depressions.

## MATERIALS AND METHODS

### Initial case

**Figure 1:**
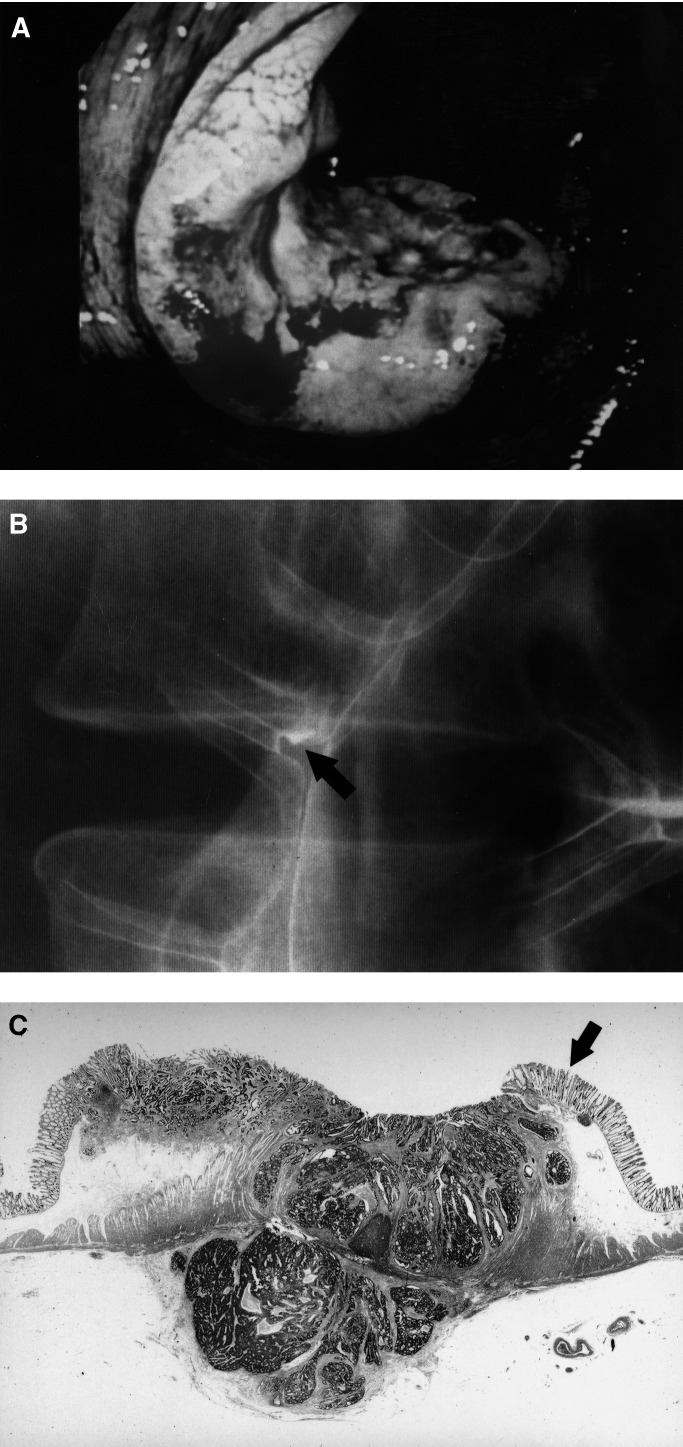
Findings in the presented case. (**A**) Colonoscopy. The tumour was relatively low and flat, with shallow central depression. (**B**) Magnified view of previous barium enema radiograph obtained 1 year previously. A small pool of barium 4 mm in diameter was present in the left portion of the transverse colon (arrow). (**C**) Low-power view of histologic findings in the lesion. At its border, the cancer was covered with normal mucosa accompanied by muscularis mucosa (arrow). This lesion was classified as showing nonpolypoid growth (NPG) type.

In October 1996, a 53-year-old woman was found to have occult blood in her stool during screening for colorectal carcinoma. No abnormality was found in an air-contrast barium enema radiograph. She had no family history of hereditary predisposition to colorectal carcinoma. In October 1997, she underwent a second examination for stool occult blood. Since the result again was positive, colonoscopy (Figure 1A

) was performed. An advanced carcinoma of 2.5 cm in diameter was discovered in the transverse colon. The original barium enema radiographs were carefully re-examined for evidence of the carcinoma. A small pool of barium, 4 mm in diameter, was seen in the location where the advanced carcinoma subsequently was detected (Figure 1B). Doubling time was estimated to be 2.4 months. Histologically, the resected tumour was a moderately differentiated adenocarcinoma infiltrating through the muscularis propria (Figure 1C) with metastases to regional lymph nodes (stage III, T3N1M0). This carcinoma was classified as having a nonpolypoid growth (NPG) pattern according to our previously published criteria (Kaneko *et al*, 1998). While no frameshift mutation of the APC gene and no Ki-*ras* mutation were present, a p53 mutation was found in exon 7 (codon 244; base change, GGC to GAC; amino-acid change, Gly to Tyr). This mutation was not located within one out of six known ‘hot spots’ for p53 mutations. No MSI was evident.

### Patient selection and data collection

Between April 1996 and March 1998, a total of 132 consecutive patients with invasive colorectal carcinomas underwent surgical resection at Showa University School of Medicine, Tokyo. Since the advanced carcinoma in the index case that originated from a depressed lesion represented stage III disease with pT3, we decided to select stage II and III carcinomas with pT3 from these cases. In all, 18 patients were excluded as the cancers were stage I, and four with liver metastases were also excluded from the study. Three patients with synchronous cancers of the colon were excluded because growth pattern and genetic alterations in such lesions could differ from that of sporadic carcinomas. None of the patients had a hereditary predisposition to colorectal carcinoma or other malignant disease. The final study group consisted of these 107 sporadic stage II or III colorectal carcinomas. Pathological features were evaluated by a pathologist at our hospital. Tumour size and location were determined from operative reports, and from clinical and pathologic data where applicable.

### Classification criteria for PG and NPG

**Figure 2:**
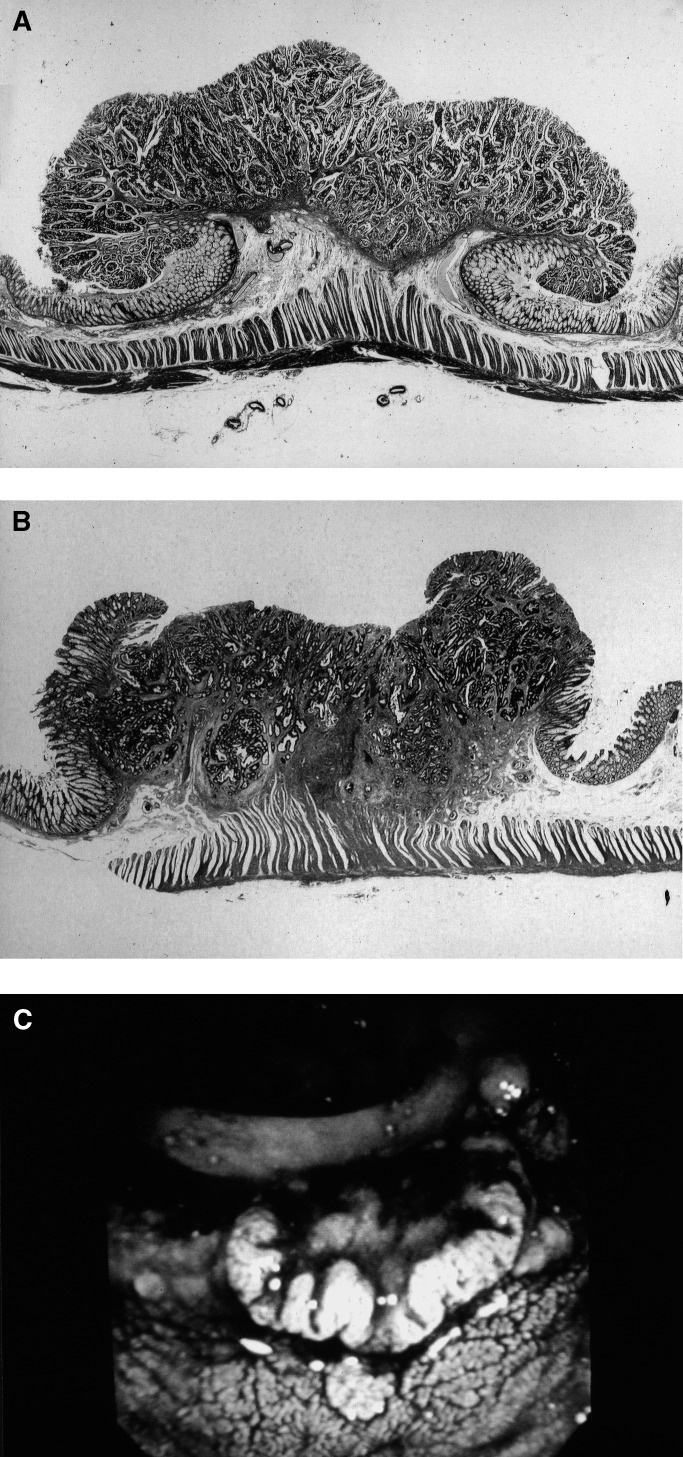
Low-power views of histologic findings in polypoid growth (PG; panel **A**) and nonpolypoid growth (NPG; panel **B**), type of carcinomas infiltrating to the submucosa, including endoscopic correlation for NPG (**C**). (**A**) At the border of the lesion, the cancer rises abruptly from normal mucosa. (**B**) At the border of the lesion, the cancer is covered with normal mucosa accompanied by muscularis mucosa. (**C**) Endoscopic view of NPG carcinoma. The lesion shows relatively little elevation, and has a central depression.

Some minute nonpolypoid intramucosal neoplasias have been reported to transform to nonpolypoid invasive cancers (Minamoto *et al*, 1994; Matsumoto *et al*, 1995; Fujiya and Maruyama, 1997). In our previous study, almost all of NPG carcinomas infiltrating the submucosa were macroscopically flat and depressed carcinomas (Kaneko *et al*, 1998). We thought that PG and NPG carcinomas infiltrating the submucosa would progress PG and NPG carcinomas infiltrating beyond the submucosa. The histologic interface between the carcinoma and the surrounding normal epithelium was carefully studied. Depending on appearance, the carcinomas were classified as having a polypoid growth (PG) or nonpolypoid growth (NPG) pattern (Figure 2A–C

). Details of this assessment have been described previously (Kaneko *et al*, 1998). Briefly, formalin-fixed surgical specimens were cut into blocks 5 mm in thickness and embedded in paraffin for sectioning. The appearance of the junction between carcinoma and normal mucosa was assessed in four or five locations for each carcinoma after sections were stained with haematoxylin and eosin (H and E). A pathologist (MK) assigned each cancer to the PG or NPG group. In carcinomas with a PG pattern, the malignant tumour rose abruptly from adjacent normal mucosa. In contrast, carcinomas with an NPG pattern showed a junction covered by normal mucosa and muscularis mucosa. The mucosa covering the junction either exhibited normal tissue or contained atypical cells.

### Analysis of gene mutations

DNA samples were extracted from tissue obtained at operation and frozen at −80°C until use. Ki-*ras* codon 12 and 13 mutations were analysed using polymerase chain reaction–single-strand conformation polymorphism (PCR–SSCP). To facilitate comparison with our previous data, the primers and PCR conditions for analysis of Ki-*ras* mutations were the same as those described previously (Sugano *et al*, 1993; Kaneko *et al*, 1998; Kaneko *et al*, 2000).

Mutations of the APC and p53 genes were analysed by fluorescence-based PCR–SSCP analysis with low-pH buffer (Kukita *et al*, 1997) using an ALF Express DNA sequencer (Amersham Pharmacia Biotech, Uppsala, Sweden) (Makino *et al*, 2000). Multiple overlapping primers were used to generate 250- to 400-bp PCR products spanning codons 1256–1640 of the APC gene; this site is known as a mutation cluster region (MCR), since a significant proportion of mutations cluster in a small region of exon 15. The reported prevalence of somatic mutations in the MCR ranges from 45 to 60% in colorectal tumours to 100% in desmoid tumours (Miyoshi *et al*, 1992; Miyaki *et al*, 1993). Based on these observations, we chose to screen for APC mutations in the MCR in our study. Primers G, H, and I in exon 15 of the APC gene were used as described previously (Groden *et al*, 1991). PCR was performed for 30 cycles (94°C for 30 s, 55°C for 30 s, 72°C for 1 min) with primer G, and for 30 two-step cycles (95°C for 20 s, 65°C for 2 min) with primers H and I. The primers and PCR conditions for exons 5–8 of the p53 gene were the same as those used previously (Makino *et al*, 2000). Furthermore, MSI (Boland *et al*, 1998) of TGF-*β*RII, BAT 26, BAT 40, D2S123, and D13S175 was analysed by fluorescence based-PCR–SSCP analysis with low-pH buffer using the ALF Express DNA sequencer. High instability (MSI-H) was defined by a novel bandshift or allele in at least 30% of microsatellite loci tested when compared to non-neoplastic tissue from the same patient (Boland *et al*, 1998). Low instability (MSI-L) designated cases with over 0% but less than 30% of markers showing a novel allele. The 5′-terminus of each primer was labelled with Cy5 dye (Amersham Pharmacia Biotech). A measure of 50 ng of DNA was used for the PCR reaction. Amplified PCR fragments were analysed by the ALF Express DNA sequencer with 5% polyacrylamide gel (acrylamide : bis/49 : 1) containing 1 × TME (30 ml Tris base, 36 mM 2[*N*-morpholino] ethanesulphonic acid (MES) and 1 mM EDTA, pH 6.8). As described previously (Makino *et al*, 2000), mutations were analysed with the Fragment manager program; shifted peaks were considered to represent DNA fragments that contained a mutation. DNA sequencing of samples that showed shifted peaks was carried out as described previously (Makino *et al*, 2000). A clear peak shift could be detected in a sample containing 10% mutant DNA, and sensitivity of PCR–SSCP under our conditions was 100% compared with DNA sequences as described previously (Makino *et al*, 2000). Direct sequencing was performed in cases that were negative by SSCP, since the presence of missed mutations in the SSCP-negative cases could not otherwise be ruled out. Sample collection and gene analysis in this study were approved by the Human Ethics Review Committee of Showa University School of Medicine.

### Statistical analysis

Data used for classifying growth patterns of colorectal carcinomas into the PG and NPG patterns were not considered in the genetic and clinicopathologic analyses; to avoid bias, these data were uncoded only afterward. The significance of differences in proportions was assessed by the χ^2^-test, Fisher's exact test, or the Wilcoxon rank-sum test. Multivariate analyses were performed using a multiple logistic regression. *P*-values less than 0.05 were considered to indicate significance.

## RESULTS

As shown in Table 1

**Table 1:** Clinicopathologic characteristics of PG and NPG carcinomas

	**PG carcinoma (*n*=86)**	**NPG carcinoma (*n*=21)**	***P*-value**
*Age, years*			
Mean (range)	64.7 (42–83)	65.1 (43–82)	NS
			
*Gender*			
Male	44 (51%)	11 (52%)	NS
Female	42 (49%)	10 (48%)	
			
*TNM classification*			
Stage II	37 (43%)	8 (38%)	NS
Stage III	49 (57%)	13 (62%)	
			
*Tumour location*			
Right	31 (36%)	8 (38%)	NS
Left	55 (64%)	13 (62%)	
			
*Tumour differentiation*			
Well	50 (58%)	8 (38%)	NS
Moderately	26 (30%)	9 (43%)	
Poorly	10 (12%)	4 (19%)	
			
*Size, mm*			
Mean (range)	55 (25–80)	33 (10–55)	<0.0001
			
*Lymph node metastasis*			
Presence	49 (57%)	13 (62%)	NS
Absence	37 (43%)	8 (38%)	
			
*Vascular invasion*			
Presence	64 (74%)	20 (95%)	0.0370
Absence	22 (26%)	1 (5%)	

PG = polypoid growth type; NPG = nonpolypoid growth type; NS = not significant (*P*⩾0.05).

, the incidence of vascular invasion in NPG carcinomas was significantly higher than that in PG carcinomas. NPG carcinomas were significantly smaller than PG carcinomas. Mean age, gender, location of tumour, tumour differentiation, lymph node metastasis, and proportion of stage II and III did not differ significantly between PG and NPG carcinomas.

As shown in Table 2

**Table 2:** Genetic changes in PG and NPG carcinomas

	**PG carcinoma (*n*=86)**	**NPG carcinoma (*n*=21)**	***P*-value**
*APC mutation*			
Presence	29 (34%)	5 (24%)	0.3818
Absence	57 (66%)	16 (76%)	
			
*Ki-ras mutation*			
Presence	31 (36%)	0 (0%)	<0.0001
Absence	55 (64%)	21 (100%)	
			
*p53 Mutation*			
Presence	48 (56%)	12 (57%)	0.9124
Absence	38 (44%)	9 (43%)	
			
*MSI*			
Presence	14 (16%)	3 (14%)	0.9806
Absence	72 (84%)	18 (86%)	

PG = polypoid growth type; NPG = nonpolypoid growth type; MSI = microsatellite instability.

, Ki-*ras* mutation was found in 36% of PG carcinomas, while no mutation was found in NPG carcinomas. Differences from the wild-type sequence were as follows: Gly to Ala, 3 (10%); Gly to Gly, 0 (0%); Gly to Cys, 2 (6%); Gly to Ser, 1 (3%); Gly to Val, 14 (45%); Gly to Asp, 10 (32%); and Gly to Arg, 1 (3%). The frequency of Gly-to-Val mutation at codon 12 was high in PG carcinomas (14 out of 31, 45%).

Prevalence of an APC mutation did not differ significantly between PG and NPG carcinomas (Table 2). As shown in Table 3

**Table 3:** Mutation patterns of APC and p53 genes in PG and NPG carcinomas

	**PG carcinoma**	**NPG carcinoma**	***P*-value**
*APC mutation*			
Type	29 (100%)	5 (100%)	
Nonsense	9 (31%)	4 (80%)	0.0189
Missense	1 (3%)	1 (20%)	
Frameshift	19 (66%)	0 (0%)	
			
*p53 Mutation*			
Type	49 (100%)^*^	12 (100%)	
Nonsense	5 (10%)	3 (25%)	0.1805
Missense	35 (71%)	5 (42%)	
Frameshift	6 (12%)	4 (32%)	
Splice site	2 (4%)	—	
Silent	1 (2%)	—	
Hot-spot mutation			
Presence	18 (37%)	0 (0%)	0.0124
Absence	31 (63%)	12 (100%)	
			
*MSI*			
Degree	14 (100%)	3 (100%)	
MSI-H	7 (50%)	1 (33%)	0.8808
MSI-L	7 (50%)	2 (67%)	

PG = polypoid growth type; NPG = nonpolypoid growth type; MSI = microsatellite instability; MSI-H = high instability (two or more out of five loci); MSI-L = low instability (one out of five loci); ^*^ = 49 mutations in 48 PG carcinomas (one case having two mutations, one nonsense, and one frameshift).

, the frequency of frameshift mutation was higher in PG carcinomas (66%) than in NPG carcinomas (0%). Mutation types of the APC gene in PG carcinomas differed significantly from those in NPG carcinomas.

Overall prevalence of a p53 mutation did not differ significantly between PG and NPG carcinomas (Table 2). As shown in Table 3, types of mutation of the p53 gene in PG carcinoma did not differ significantly from those in NPG carcinoma. However, involvement of hot spots differed greatly. Of 49 mutations in PG carcinomas, 18 (37%) were found in hot spots at codon 175 (four patients), 245 (three patients), 248 (seven patients), 273 (three patients), and 282 (one patient); no patient had a mutation at codon 249. In contrast, the 12 p53 mutations in NPG carcinomas were not found at hot spots. The prevalence of p53 mutations at hot spots thus was significantly higher in PG carcinomas than in NPG carcinomas (Table 3). Direct sequencing performed on all cases that were negative by SSCP did not disclose any mutations. The sensitivity and specificity of the PCR–SSCP assay for the mutations were 100 and 90%, respectively.

No MSI was found in non-neoplastic tissue from any of the 107 patients. The frequency of MSI in PG carcinomas did not differ significantly from that in NPG carcinomas (Table 2). The frequency of patients showing instability at all five loci was five out of 17 (29%); four out of five loci, no patient (0%); three out of five loci, one patient (6%); two out of five loci, two patients (12%); and one out of five loci, nine patients (53%). All five patients with an abnormality of TGF-*β*RII also had abnormalities at the other four loci, and all five patients had PG carcinomas. Of eight MSI-H tumours, seven (88%) were PG carcinomas. Of three NPG carcinomas with MSI (Table 3), one was MSI-H (three of five loci); the remaining two were MSI-L (one out of five loci).

Multivariate analyses were performed using multiple logistic regression. Tumours were classified as PG or NPG carcinomas, and these groups were compared concerning patient age, gender, clinical stage, tumour size, vascular invasion, tumour location, tumour differentiation, APC mutation, Ki-*ras* mutation, p53 mutation, and MSI. Significant differences were evident for tumour size (*P*=0.00002) and vascular invasion (*P*=0.02417). Presence of a Ki-*ras* mutation showed a significant difference between PG and NPG carcinomas in univariate analysis (*P*<0.0001). This difference was not evident by multiple logistic regression (*P*=0.99995) because no Ki-*ras* mutation was found in NPG carcinomas.

## DISCUSSION

This study was prompted by the apparently rapid progression of a minute depressed lesion to an advanced nonpolypoid growth carcinoma within only 1 year. We propose that colorectal neoplasias with a central depression develop into carcinomas with a nonpolypoid growth pattern.

In our previous prospective study in which 2720 consecutive patients undergoing total colonoscopy were examined for flat lesions, a Ki-*ras* mutation was not found in any intramucosal flat lesion with a smooth surface (Kaneko *et al*, 2000). In another previously published study, we found no Ki-*ras* mutation in NPG carcinomas infiltrating the submucosa (SM) (Kaneko *et al*, 1998). SM-NPG carcinomas showed neither a coexisting adenomatous component nor a Ki-*ras* mutation. In contrast, 75% of SM-PG carcinomas had a coexisting adenomatous component, and 44% had a Ki-*ras* mutation. We reported that SM-PG carcinomas arose from protruding adenomas according to the adenoma–carcinoma sequence, while the growth pattern of SM-NPG carcinomas was different. Vogelstein *et al* (1988) proposed that Ki-*ras* mutation is an early event in the adenoma–carcinoma sequence with Ki-*ras* mutational status possibly determining development of an exophytic polypoid adenoma. Our results suggest that in contrast a Ki-*ras* mutation might not be present in the NPG carcinoma pathway even when the lesion is advanced. Our previous and present studies indicate that Ki-*ras* mutation is not related to genetic progression in morphologically distinct nonpolypoid lesions containing NPG carcinomas.

Our study showed that NPG carcinomas were significantly smaller than PG carcinomas. In spite of this, vascular invasion was significantly more frequent in NPG carcinomas than in PG carcinomas. It is likely that the biologic behaviour of NPG carcinomas is more aggressive than that of PG carcinomas. Many reports have analysed that flat and depressed lesions have a higher malignant potential than polypoid lesions, since flat and depressed lesions readily infiltrate deeper layers despite their small size (Crowford and Stromeyer, 1983; Wolber and Owen, 1991; Lanspa *et al*, 1992; Jaramillo *et al*, 1994; Rubio *et al*, 1994; Kudo *et al*, 1995a, 1995b; Mitooka *et al*, 1995; Yokota *et al*, 1995; Fujii *et al*, 1998; Hart *et al*, 1998; Rembacken *et al*, 2000; Saito *et al*, 2001). A greater malignant potential in NPG carcinomas than in PG carcinomas is analogous to flat lesions containing depressed areas having greater malignant potential than polypoid adenomas.

Mutation patterns in the APC gene differed significantly between PG and NPG carcinomas. More than 700 somatic mutations of the APC gene have been reported to date in different tumour types, with more than 90% of these mutations being observed in colorectal adenomas according to Laurent-Puig *et al* (1998). The same authors reported that most of these somatic mutations led to truncation of the APC protein, either by frameshift mutation (62%) or by nonsense mutation (34%). The proportion of frameshift and nonsense mutations in the report of Laurent-Puig *et al* was similar to that in PG carcinomas in our study. In our study, however, no frameshift mutation was found in NPG carcinomas. Other group has reported similar findings (De Benedetti *et al*, 1994). De Benedetti *et al* (1994) reported that frameshift mutation of APC gene was predominant in polypoid adenomas, with APC mutations participating in progression of exophytic adenomas. In contrast, Umetani *et al* (2000) have reported that the frequency of APC mutation in nonpolypoid adenomas was significantly lower than that in polypoid adenomas, while being similar between polypoid carcinomas and nonpolypoid carcinomas. They concluded that new APC mutations are acquired at the time point representing malignant transformation during the development of nonpolypoid adenomas. We believe that APC mutations in NPG carcinomas are related to malignant transformation, since we found no significant difference in frequency of APC mutations between PG and NPG carcinomas. Yet we found no APC frameshift mutations in NPG carcinomas. If the presence of APC mutations in NPG carcinomas is indeed related to malignant transformation, we suspect that specific mutation patterns of the APC gene importantly affect the genetic pathway leading to NPG carcinoma.

With respect to the p53 gene, 6177 somatic mutations in exons 5 to 8 have been reported in different tumour types (Hussain and Harris, 1999). The overall prevalence of nonsense, missense, frameshift, splice site, and silent mutations among these 6177 mutations was 6, 78, 10, 3, and 3%, respectively. These relative prevalences were similar to those in our PG and NPG tumours. In contrast, mutation of the p53 gene has been proposed to be concentrated at hot spots (Hainnaut *et al*, 1998; Hussain and Harris, 1999). Mutations were found at one or more of six hot spots in exons 5–8 in 25–30% of large case series (Greenblatt *et al*, 1994; Hainnaut *et al*, 1998; Hussain and Harris, 1999); similarly, the prevalence in all of our cases considered together was 28%. Hot spots for somatic mutations in carcinomas represent protein alterations that provide a selective growth advantage to the cell. Many reports suggest that a hot spot can identify relationships between mutation, protein structure and function, and carcinogenesis (Hsu *et al*, 1991; Cho *et al*, 1994; Greenblatt *et al*, 1994; Tornaletti and Pfeifer, 1994). In this study, no hot-spot mutations were found in NPG carcinomas. The difference in p53 mutation location between PG and NPG carcinomas may suggest differences in function correlating with differing morphologic development.

The frequency of MSI did not differ significantly between our PG and NPG carcinomas, although most carcinomas rated MSI-H were PG carcinomas (88%). Loukola *et al* (1999) examined MSI in 402 sporadically occurring adenomas, with only six adenomas (1.5%) being MSI-H. Furthermore, five of these six adenomas subsequently proved to have arisen in subjects with hereditary nonpolyposis colorectal cancer (HNPCC). In our study, eight out of 107 carcinomas (7%) were MSI-H; most carcinomas rated MSI-H were PG carcinomas, which were considered to have arisen from polypoid adenomas. In the study of Jass *et al* (2000) MSI-H was commonly observed in dysplastic areas of serrated polyps, as was MSI-L. We believe that PG carcinomas may include most carcinomas derived from serrated adenomas.

Clinicopathologic findings in our study suggest that development of NPG carcinomas is more aggressive than that of PG carcinomas. The genetic makeup of NPG carcinomas is unique, not being based on the conventional adenoma–carcinoma sequence. We suggest that the genetic pathway of NPG carcinoma is distinct from that of PG carcinoma even in early stages of carcinogenesis. The morphologic characteristics of carcinomas originating from nonpolypoid lesions may reflect differences in genetic pathways giving rise to cancers. Furthermore, some minute nonpolypoid neoplasias have reported previously to transform to nonpolypoid cancers manifesting rapid growth (Minamoto *et al*, 1994; Matsumoto *et al*, 1995; Fujiya and Maruyama, 1997). If we assume that some flat or depressed lesions rapidly transform into advanced nonpolypoid growth carcinomas, a maximum of 20% of colorectal carcinomas could be expected to progress in this manner.
